# What Is Disease?

**Published:** 1883-06

**Authors:** W. Godfrey Dyas


CHICAGO MEDICAL

### JOURNAL & EXAMINER.

VOL. XLVI.—JUNE, 1883.—NO. 6.

### Original Communications.

ARTICLE I.

WHAT IS DISEASE? BY PROF. W. GODFREY DYAS, F. R. C. S. (Inaugural Address delivered at the Woman's Medical College, October, 1878.)

On an occasion like this, it may be expected that I should formally inaugurate the course, by a history of the rise and progress of medical science; range through, from remote periods, the intervening space to the present; detail views long since abandoned, and which have given place to others destined to share the same fate;—that I should traverse regions of error, unillumined by a single ray of collateral science, and having toiled through the literary desert, felicitate myself that I had at length reached a point where sterility and darkness gave way to the gradual but steady unfolding of truth, as read in the bright light of day. But, even if my inclination prompted the inquiry, it would not be consistent with the plan I propose to pursue, which necessarily restricts me in the choice of my materials; nor would it conduce to your interests, which may be much better served than by an enumeration of unprofitable theories. Yet I do not intend to say that an investigation of this kind would be wholly devoid of interest to those who have time and leisure to indulge in the review of past opinions, nor to affirm that many a literary gem may not reward a search among even the rejected matter and rubbish of our predecessors. The question which now, at the very threshold of a year of literary labor, chiefly interests us, is one upon the correct answer to which, much of your future success will depend. It involves principles of the deepest importance to you, when you become practitioners, and to your patients in their relation to you in that capacity, and it is one on which you should have well defined opinions, before you presume to deal with it practically. What is disease? is the subject of our inquiry. Is it an entity, superadded to the organism, and interfering with its normal functions? Or is it an effort at restoration of impeded or deranged functions, with a tendency to be salutary? Should the organisim be capable of maintaining its conditions for existence during a rough, but well-meant intervention? Or is it simply a phase of life; is it the complement which, with what we call health, makes up and completes the idea of life? Should we adopt the first view, we look on disease as merely something that preys upon the organism, which, in that case, would be restored to its pristine health if this enemy were removed; and hence, we have numberless popular expressions based on this supposition, which doubtless emanated from our profession. This was the prevailing opinion. Subsequently, a more correct view was entertained. It consisted in looking on the morbid phenomena as the vital expressions of suffering organism; the result of a vital reaction to the injury sustained, and hence it was inferred that the duty of the physician was to assist this vital reaction, and aid it as a normal and physiological action. This theory embodied some truth, but the inference deduced was faulty. The first theory led to the employment of powerful measures, often more detrimental than the disease which they were intended to combat. The second favored an encouragement of disease. If, then, we wish to get a correct view of disease, we must look at it and health as two separate phases of the phenomena we term life; and we must consider the relations that always exist between living organized bodies, and the agents which influence them in the manifestation of these phenomena.

As we proceed from the simplest to the most complicated organization, the susceptibility to the action of a stimulus is the cause of living action, through all the states of animal life. It is simply a mode of being of matter, depending on the placing of molecules in certain relative positions. It is not necessarily dependent on the agency of a nervous system; we see it in animals in which such a system does not exist. We see it present itself under various forms, as regards muscle, gland or nerve, each receiving an impression when subjected to a suitable stimulus. In the more elaborate organisms, we observe a coördination of these several impressions, uniting them in consent and accordance, principally, if not altogether, through the intervention of a nervous system, and forming, as it were, one consentient whole of the several and apparently dissimilar organs. So long as, during the never-ceasing change of the molecules of matter, vital reactions take place with ease and accuracy, there is health; the moment they cease to observe conformity to the laws of their existence (which are as much impressed on them as the attraction of gravity on dead or inorganic matter), and that they are marked by want of exactness, owing to the action of abnormal stimuli, there is disease. Beyond this, we cannot carry our inquiries. It is idle to attempt to reason as to the determining causes of these actions. They lie far beyond the range of human contemplation. The several stimuli themselves are subordinated to our reason. On them, we may legitimately speculate.

Heat, air and aliment—the necessary stimuli in the production of living actions, may range within variable, yet, as regards individual organizations, certain bounds, consistently with health. Beyond these, they give rise to a manifestation of a more prolonged and intensive series of changes, with a view to the maintenance of the well-being of the organism under the superinduced and novel condition. These changes, then, constitute what we call disease. They tend to the restoration of health, and only fail from the powers of the organism being inadequate to the completion of the necessary cycle of organic changes. Under these circumstances, the duty of the physician is twofold. On the one hand, he has to maintain the powers of the organism ; on the other, to modify the newly developed process of nature. His capability of effecting the former cannot be disputed. It is a matter of daily observation, proved by supplying or withholding the normal stimuli necessary to the continuance of the several functions. But can we modify a newly developed process of nature? Analogy would lead us to suppose so; for as nature, under its other numerous phases, admits of modification at the hands of art, it is reasonable to infer it may not find an exception in not being amenable to our art, when under the well-directed and skillful guidance of science. Besides, what reason in this respect suggests, seems confirmed by the daily experience of the profession in the action of certain medicines, whose efficacy is incontrovertibly established. None can question the efficacy of quinine in malarious disease, nor of mercury and iodide of potash. It is true, that in some diseases our efforts are restricted within narrower limits. In the specific fevers, for instance, that run a definite course, we have little else to do than, as Watson expresses it, "to see that nature has fair play, to redress some untoward deviations from the regular course, or to facilitate and fortify the natural recuperative efforts." Yet, in fulfilling these general indications, we may materially contribute to a favorable result, by subduing the morbid temperature through the medium of cold applied generally and locally, and by sedative doses of quinine and bromide of potassium.

Whilst thus glancing at that part of medicine termed therapeutics, I cannot avoid acknowledging how defective it is, when compared with our diagnosis and prognosis of disease, notwithstanding all the time and learning that have been bestowed in the discovery and application of remedies. Yet, on reflection, we cannot help seeing that, from the very nature of the subject, it cannot be otherwise. In dealing with it we have not, as in the diagnosis of a case, an assemblage of signs and symptoms to estimate individually and in combination, and compare together, supported as they are by a knowledge of morbid anatomy and pathology. On the contrary, to a great extent, we must be content to glean in the field of empiricism, where, though the laborers are many, their capacities for judging of the materials they gather, differ as much as the minds and dispositions they bring to the task. Again, whilst administering medicines for some particular malady, complications previously latent will come to the surface, frequently, it may be, developed by our well-meant efforts to remove the original disease, and give rise to more serious morbid phenomena than those they were intended to combat; thus leading us astray in regard to the efficacy of the means we use. Besides, the features of a disease are constantly varying, those of any two cases never being exactly alike, and thus we never, in any two consecutive cases, apply our remedies under precisely the same conditions. Another source of error in therapeutics, must be attributed to the loose mode in which we daily express ourselves, using terms that imply our acquiescence in theories, some long since exploded, and others, though still entertained, yet having no real foundation either in facts or sound deduction. We speak of the elimination of poison, and the purifying effects of certain remedies; and if we limit these terms to operations on the organism, where mineral poisons are in question, I will not dispute the propriety of the language used. But I deny that it is suitable when applied to diseases that result from organic poisons. In this latter case, a permanent effect has been left, that militates with the notion that a something has been removed, leaving the organism as it was before—as if simply a poison had been cast out. Again, we read of zymotic diseases. This dogma of zymosis rests on uncertain grounds. A zymotic disease is one supposed to be produced by some morbific principle acting on the system as a ferment. Do we see in the phenomena of fever anything characteristic of fermentation? Certainly not. Besides, the theory is based on the assumption that the first morbid movement in fever commences in the blood. It may be so. But where is the proof? In such cases, the first intimation of perverted function is supplied by the nervous system. When we speak of the initial chill of fever, we use correct language; we thus express the disturbed function of the nervous centers, that ushers in the sequence of morbid and specific processes that constitute the disease.

But, though I acknowledge therapeutics to be the most vulnerable point of our position, yet let me not be supposed to have any intention of conveying the false notion, that the diagnosis of disease is brought to perfection. That would imply a perfect physiology, and still more a pathology as well established as a fixed science. These desiderata, I need scarcely say, are unattainable. In some forms of disease, where we have physical signs coming to the aid of symptoms; where, in a word, the subjective and the objective are conjoined, as in affections of the lungs and heart, we have unusual facilities, when guided by a ripe experience and sound judgment, to form a creditable and satisfactory diagnosis. The same observation will justly apply to certain instrumental appliances which we have more recently availed ourselves of, to aid us in our researches. The sphygmograph, the laryngoscope, the aspirator, the thermometer, have now their place, and to considerable advantage, in the armamentarium of the practitioner. But, on the other hand, in other forms of disease, in cerebral affections, for instance, where we apparently have opposite conditions of a part, producing the same morbid phenomena, different lesions causing the same symptoms, the seat of disease being the same, we are compelled to acknowledge we have much still to learn, before we can in every case, with perfect satisfaction to ourselves, or the greatest amount of profit to our patients, proceed to treatment.

Whoever, for a moment, considers the varying and uncertain estimation in which remedial means are from time to time held, will hesitate to give his adhesion to views in connection with them, merely because their employment is due to the capricious taste of the day. He must examine for himself, as well as entertain the suggestions of authority, uninfluenced by the ruling of any arbitrary and passing fashion; and he must endeavor, in each case, to refer their action to some sound scientific principle. And now, before I proceed further, let me impress on you the fact, that anything in our profession that is not based on science, must be received with suspicion. I do not mean to say, that I would reject a remedy, the undoubted efficacy of which our individual experience and that of others testify to, until we can satisfactorily and on scientific grounds receive it, until, in short, we can place it in a suitable niche in the temple of science. Nor should we ignore morbid manifestations as signs and symptoms of disease, until we can interpret them according to the notions of our philosophy, and the doctrines of our schools. But on the other hand, we must guard against a facile assent to new views not in accordance with science—often in positive opposition to its teachings. We must avoid being "carried about by every wind of doctrine." At all times in our profession, as well as in concerns of more momentous import, innovations, "falsely termed science," have crept in, which have charmed, by their novelty, half-educated people, whose erudition, scanty and slender at the best, was not directed by a sound or logical mind. For the most part their success has been fleeting and evanescent, scarcely surviving the term of their novelty. Other fallacies have, however, for a longer time succeeded in enchaining the minds of men, introduced as they have been, with all the circumstances of plausible argument, and the semblance of being reduced to a principle. The most noted of these owes its importance to an apostate member of our profession, who, being in his early days unsuccessful in practice, revived a dogma that had been enunciated and maintained in the 6th century by Gregory the Great. The dogma *similia similibus curantur* therefore lacks even the quality of novelty. This exploded tenet—this fallacy of ungrounded persuasions can easily be discovered and unmasked by the enquiry of a mind of even moderate comprehension, and can be exposed as either a miserable delusion or a culpable imposition. The advocates of orthodox medicine do not mean to assert that their own system is free from defects, but they do not hesitate to state that they rest its merits on the broad basis of acknowledged science. It is not cramped by any spurious doctrine, but invokes the aid of all knowledge, whether obstruse or obvious, and owing to this it has had the sanction of the learned for centuries.

For didactic purposes it has always been desirable to have some system of nosology. Distinguished physicians have at different times laid down systems, so-called, of disease all of which have by their successors been found defective and have been replaced by others not more fortunate in securing the favor of the profession. When you consider the basis on which each system was raised; that such was in accordance with the received opinions of the day in regard to science, and that this in every department was undergoing modification from additional light shed on it, you cannot expect that the superstructure could remain together on a foundation so unstable. We cannot, nevertheless, dispense with order, and in furtherance of this we must have some system—some assemblage of the constituent parts of a subject into a general whole, wherein the connection or mutual dependencies may be seen without much effort of the understanding. With this view the best arrangement we can adopt will always be that founded on a physiological basis, in which the diseases of the respective functions of the animal frame are connected in classes derived from those functions, and follow each other in the order in which physiologists have usually treated of them. After all, Nature, infinitely varied, will not bend herself to an intelligence which can but assign bounds and make divisions. You cannot reunite the double advantage of requisite precision in details, and at the same time clear philosophical views as regards the several phenomena, morbid and normal of the organism. You may remark that in medical literature we draw largely on the Greek language for a nomenclature of medicine, and we assume that those who enter our profession have not only already had some acquaintance with the rudiments of the ancient classics but have acquired a competent knowledge of that branch of learning. I have often heard men—uneducated men, always—say, "What have the classics to do with the practice of medicine?" I would reply, certainly none directly—much, however, indirectly. And I take this opportunity to express my deep regret at the great falling off, of late, in the preliminary education of the alumni of our schools. To such an extent has this decadence of our profession been carried, that some of our graduates have been unable to translate their own diplomas, and that many cannot muster sufficient knowledge of their mother tongue to publish their cases. The time was when a physician from his superior education and resulting refinement, was the fit associate of all that were elevated in society; the literary title affixed gave him access to and acceptance by those distinguished by position and culture. Now the same title is often calculated to excite a smile, conferred as it is so frequently on men who are below the lowest grade of mechanics. The greatest evil to which, in the present day, our profession has been subjected, is the annual intrusion into its ranks of hordes of uncouth and uncultured persons. How far this disregard of preliminary education is beneficial to colleges or calculated to increase the fund from fees paid for diplomas, is just now foreign to my subject.

Our profession is now so degraded that it is rare to find men capable of reading any medical work not written in their vernacular language. In this city containing so many Europeans from countries of various tongues, it is necessary for a physician to be acquainted with the Latin language. It has been for centuries the great vehicle of knowledge amongst the learned of civilized nations. It has conveyed to us the traditional literature of our calling. Some of our best apothecaries are foreigners, all of whom possessing a qualification from the licensing bodies of their respective countries have a competent knowledge of Latin, and can, with ease, comprehend our wishes expressed in that language, but who often find much difficulty in understanding us when we write in English. It is also desirable that you should have at least such knowledge of German and French as will enable you to read, in the original, works published in these languages. The perusal of a book in the words of an author, is generally a very different thing from a translation of the same, no matter how excellent the version may be.

In conclusion, I must observe, few studies take so wide a range as ours. To acquire a perfect knowledge of physiology—if such be possible—we must be acquainted with the properties of the material world by which we are surrounded, and of which our frames are formed. We must know the laws that regulate the forces of matter; for we are not exempt from these laws. All tho subtle agencies of nature, gravitation, electricity, heat and light, all influence more or less and modify our condition. We may be well versed in the anatomy of the eye, but unless we know something of the laws which govern the refraction of light, we are unable to appreciate the adaptation of means to ends in the construction of the organ. Again, we may have a perfect knowledge of every structure entering into the formation of the ear, but unless we know the cause, nature and phenomena of sound, our anatomical knowledge is a barren and unprofitable possession. The same observation will apply to the larynx—that wonderful apparatus for the production of the tones and inflections of the voice.

I now close with the words of a distinguished member of our profession, who has recently passed away from us: "In contemplating the living man, we regard not only his wonderful material fabric, but we see him endowed with faculties, which the laws of nature cannot explain—a creature of intelligence, with a moral sense, judging of right and wrong, and with a hope of a future and higher existence. And since we find that in the material framework, every part is perfectly adapted to its end, and to man's wants—the eye for sight and the ear for hearing,—and is perfectly suited both to the material things around, and to man's intellectual and moral nature; that considered in reference to the world without and the world within, it is perfect and complete, we may naturally infer that the intellectual and moral powers and capacities which make up the inner man—which are perfectly suited like the bodily organs to the material things around, but which are confessedly unsatisfied with their relations to them—that these faculties and powers have other and higher relations in a different sphere; that there is a completeness and a wise purpose in every faculty of the mind, as there is in every part of the bodily frame, and that the desire therefore of a future and higher existence has not been implanted in us in vain, and that the promise of it given to the heart will not be broken."

